# Evaluation of Taste Perception and Olfactory Function in Adolescents in Relation to the Duration of Type 1 Diabetes

**DOI:** 10.1155/pedi/5563863

**Published:** 2025-06-04

**Authors:** Grzegorz Sobek, Paweł Jagielski, Mariusz Dąbrowski, Artur Mazur

**Affiliations:** ^1^Faculty of Health Sciences and Psychology, Collegium Medicum, University of Rzeszów, Rzeszów, Poland; ^2^Department of Nutrition and Drug Research, Institute of Public Health, Faculty of Health Sciences, Jagiellonian University Medical College, Krakow 31-066, Poland; ^3^Faculty of Medicine, Collegium Medicum, University of Rzeszów, Rzeszów, Poland

**Keywords:** adolescents, smell, taste, type 1 diabetes

## Abstract

**Background:** Olfactory dysfunction may be one of the clinical symptoms of neuropathy in diabetics. It is also known that taste and smell disorders in diabetes may impact dietary adherence and, consequently, good glycemic control. The study aimed to investigate taste perception and olfactory function in adolescents with type 1 diabetes (T1D) compared to the control group.

**Materials and Methods:** The study was conducted on patients aged 11–15 from south-eastern Poland with T1D in the diabetes clinic of the 2nd Department of Pediatrics, Endocrinology, and Diabetology of the Provincial Clinical Hospital No. 2 in Rzeszów. Taste strips (sweet, salty, sour, and bitter), *U*-Sniff sticks (12 selected smells), and a filter paper strip impregnated with n-propylthiouracil (PROP) were used.

**Results:** No significant differences were observed between both the groups in the taste tests, except for the sweet taste test. The sweet taste test scores were higher for diabetes, for whom the median score was 4 (3.0–4.0), than for the control group, for whom the median score was 3.0 (3.0–4.0), (*p*=0.0001). These results mean that diabetics were more sensitive to sweet taste. We also found no significant differences between T1D and the control group of the *U*-Sniff test. However, significantly lower *U*-Sniff test scores were seen in adolescents with diabetes of more than 10 years. Median *U*-Sniff test scores for people with diabetes for 10 years were 11 (10.0–12.0) while in the control group median was 10.0 (9.0–12.0), (*p*=0.0370). The analysis also showed that adolescents suffering from long-term T1D more often incorrectly identified bitter tastes than healthy adolescents.

**Conclusion:** The duration of diabetes is important regarding changes in taste perception and olfactory function in adolescents with diabetes.

## 1. Introduction

Type 1 diabetes (T1D) is a chronic systemic disease of childhood. Over time, it leads to serious complications affecting most body systems [1]. Some of the complications of diabetes have been thoroughly investigated and understood. Nevertheless, diabetes and its complications likely cause a variety of problems about which little is known. Diabetic neuropathy is a common condition in people with diabetes. Central forms of neuropathy affecting the gray matter and cranial nerves may be diagnosed in some people with diabetes. It is believed that the sense of smell dysfunction may be a clinical symptom of central neuropathy [2]. Some have also reported that diabetic peripheral neuropathy (DPN) and neuropathic pain were associated with olfactory dysfunction [3]. Interestingly, it has been suggested that diabetes comorbidities, such as liver and kidney failure, cardiovascular disease, depression, or hypothyroidism, were associated with increased risk of isolated olfactory dysfunction independent of glycemic control [4]. The correlation between taste function and diseases, such as obesity and diabetes, has garnered significant research interest recently. Taste disorders in type 2 diabetes (T2D) have been confirmed in several publications, suggesting a possible impact of this disorder on the ability to adhere to a controlled diet and, consequently, achieve optimal glycemic parameters [5]. Altered taste function has also long been reported in affected individuals with T1D. Hypogeusia has also been described and diagnosed based on assessing four basic tastes (bitter, salty, sour, and sweet) in adults with T1D [6]. Undoubtedly, recognizing the taste qualities of sweet, salty, sour, bitter, and umami is one of the most important aspects of food acceptance. Especially, the ability to perceive sweet and bitter taste is related to individual dietary choices [7–9]. There is therefore a need to determine the relationship between the development of diseases and sensory perception, as it is believed that the sensations of taste, smell, and chemesthesia can be helpful in their treatment [10]. The existing literature is primarily based on adults and fails to establish a clear taste and smell perceptions relationship with diabetes and related pathologies. However, attention was drawn to some possible association in the adult population, in which the duration of the disease may have influenced the relationship [11]. The study aimed to identify differences in sensory functions between adolescents with T1D and a control group. In individuals diagnosed with T1D, we also analyzed the potential impact of personal characteristics (gender, body mass index [BMI], breastfeeding, and presence of diabetes in immediate family) and diagnosed with celiac disease (CD) on the function of smell and taste. Research involving pediatric populations is scarce and typically focuses on very small patient cohorts. Hence, this study has the potential to make a meaningful contribution to the field.

## 2. Materials and Methods

### 2.1. Study Populations

Patients were recruited in the diabetes clinic of the 2nd Department of Pediatrics, Endocrinology, and Diabetology of the Provincial Clinical Hospital No. 2 and the Hospital of St. Jadwiga Queen in Rzeszów from October 1, 2020, until February 1, 2022. Finally, 148 children aged 11–15 were qualified for the study. The inclusion criteria for children were 11–15 years of age and parents' consent to participate in the study. All children recruited for the study had T1D for at least a year. As part of the cooperation of the University of Rzeszów with primary schools from the Podkarpackie Voivodeship, a group of 100 children selected according to age and gender was also examined as a control group. Parents of the examined children from the control group gave written consent to participate in the study. The exclusion criteria for both groups of children included the use of medications known to influence the senses of smell or taste, the consumption of antibiotics within 2 months prior to the commencement of the study as well as a runny nose in test day that affects the olfactory function Moreover, we excluded patients with implanted pacemaker and pregnancy due to contraindications for the bioimpedance test.

### 2.2. Study Measures

#### 2.2.1. Taste Function Assessment

We used “taste strips”, which are often used for diagnosing taste dysfunction. “Taste strips” (Burghart, Wedel, Germany) are filter-paper strips impregnated with a taste solution and determine sweet, sour, salty, and bitter taste scores [12]. The different taste strips with concentration of sucrose for sweet taste (0.05, 0.1, 0.2, and 0.4 g/ml); quinine hydrochlore for bitter taste (0.0004, 0.0009, 0.0024, and 0.006 g/ml); citric acid for sour taste (0.05, 0.09, 0.165, and 0.3 g/ml); and sodium chloride for salty taste (0.016, 0.04, 0.1, and 0.25 g/ml) was presented in an order of increasing concentrations in a pseudorandomized manner. In addition to the impregnated strips, strips with no taste were offered. Pseudo-randomization concerned both concentrations and tastes, including strips without taste. The test sequence was performed according to the strip manufacturer's instructions. As recommended, the patient was initially given a strip without taste and told that this was what a “clean” strip of paper tasted like. The research was carried out in optimal conditions for sensory tests. They included a previously ventilated, noise-free room with a comfortable temperature of 20°C. All study participants were tested in the morning between 8 a.m. and 11 a.m. Participants were requested to abstain from consuming food and drinks (except water at room temperature), brushing their teeth, and chewing gum for 1 h before the test. To assess taste, the participant placed strips given to him/her by the researcher in the middle of his/her tongue. The participant then discarded the strip and took a sip of bottled water to rinse the water around the mouth. Afterwards, the participants were asked to evaluate the perceived taste, indicating whether it was sweet, salty, sour, bitter, or tasteless. Scoring was based on a yes/no system for each concentration, which ranged from 0 to 4 based on the number of concentrations correctly identified. To obtain the total taste score (0–16), individual taste quality scores were summed. A higher score indicates greater taste sensitivity. According to the previous literature, a total score of less than 9 signified hypogeusia. Hypogeusia can be defined as the reduced ability to taste things. Individual test scores lower than 2 (sweet, sour, and salty taste) and lower than 1 (bitter taste) also indicate hypogeusia. For a total of four tastes, values of 9 and above are normogeusia. Individual sweet, sour, and salty taste test values of 2 or more are treated as normogeusia. In the case of bitter taste, the normative values are 1 or more [12–14].

n-propylthiouracil (PROP) test: PROP tasting is a common genetic trait found throughout the world's population. There are significant individual differences in taste sensitivity to the bitter compounds PROP and thiouracil. The TAS2R38 (T2R38) gene is primarily responsible for encoding the receptor that detects bitter taste and for distinguishing between those who perceive PROP as tasteless (nontasters) and those who experience a moderate to strong bitter taste of these chemicals (tasters) [15]. PROP is considered a paradigm gustatory stimulus in humans [16]. PROP taster status was determined by a filter paper strip impregnated with PROP (Sensonics International, NJ, United States, 20 µg/strip). Each participant was asked to put the strip on the upper surface of their tongue for about 30 s and then reported if they tasted anything (yes/no). Participants who answered “no” or responded that the strip “tastes like paper” were classified as “nontasters.” Children indicating that the strip tasted “bitter,” “sour,” “bad,” or “spicy” were classified as “tasters.” Additionally, if participants promptly removed the strip because they found it tasted “foul” or exhibited other forms of taste rejection, they were also categorized as “taster” [17].

#### 2.2.2. Olfactory Testing

We used the 12-item “*U*-Sniff” smell identification (ID) test to preliminarily assess the olfactory functions of the participants. Based on the current literature, the *U*-Sniff smell ID test is one of the tools recommended for accurate and reliable testing of the sense of smell in children [18, 19]. The ID test is used to assess the smell function in children aged 6–17 years [20]. The test results may serve to distinguish between normosmia (normal olfactory function), hyposmia (impaired olfactory function), and anosmia (residual or absent olfactory function). The test for ID involves 12 pens, each filled with a recognizable scent (coffee, apple, banana, butter, fish, cut grass, flower, lemon, onion, orange, peach, and strawberry). A four-alternative forced-choice procedure is followed for each pen, in which respondents identify each pen's odor by picking from a list of four options. In total, each participant smells 48 odor descriptors and identifies 12 of them. Scoring was based on a system of 1 for each correct answer and 0 for each incorrect answer. Therefore, the total possible score was from 0 to 12. Higher test scores signify better olfactory function. The testing procedure was the same for all participants. Large normative data sets are available for this test, which covers the different ages of the children and different countries of origin. Based on previous literature data and the manufacturer's instructions, the cut-off value for normosmia was defined as ≥10 points [20–22]. In this study, *U*-Sniff test results from 7 to 9 were classified as hyposmia, and values of 6 or less were classified as anosmia. It is important to note that the cut-off criterion mentioned above was established for this study, given that the prevalence of reduced olfactory function in children is still unknown [22].

#### 2.2.3. Anthropometric Evaluation

Height was measured three times for each participant using a portable SEA 213 stadiometer, accurate to 5 mm. Participants stood upright, wearing light attire and no footwear. The average of the three measurements was utilized in the study's analysis [23]. Body weight was determined accurately to 0.1 kg with a body composition analyzer (BC-420, Tanita, Tokyo, Japan). Participants' height and weight were assessed in a fasted state in the morning. Using the formula weight (kg)/height (m)^2^, the BMI was determined. Following this, individual BMI percentiles were calculated using age-, sex-, and height-specific charts. For percentile calculations, charts derived from the Polish project titled “Developing standards of blood pressure in children and adolescents in Poland, OLAF” were utilized [24]. According to BMI percentile values, participants were assigned to one of four categories: underweight (<5th percentile), normal weight (between 5th and 85th percentile), overweight (85th percentile and above, but <95th percentile), or obesity (95th percentile and above).

#### 2.2.4. Biochemical Analysis

Following a 10-h overnight fast, blood samples were taken at the hospital laboratory. Standard laboratory methods (Siemens Atellica, Germany) were then used to measure lipid profiles, which included total cholesterol (TC), high-density lipoprotein (HDL) cholesterol, low-density lipoprotein (LDL) cholesterol, and triglycerides (TGs). The measurement of glycated hemoglobin (HbA1c) was performed using an automated glycohemoglobin analyzer (G8 HPLC, Tosoh Bioscience, Tokyo, Japan).

#### 2.2.5. Assessment of Other Possible Personal Factors

This study's self-reported questionnaire included questions regarding general demographic information (age and gender). The questionnaire asked about cases of diabetes in the immediate family. One of the questions concerned breastfeeding. Questions were asked about the period of breast milk feeding. Breastfeeding was categorized into two groups: those who breastfed for 6 months or more and those who breastfed for less than 6 months or not at all. Additionally, questions were asked about the duration of diabetes and CD diagnosis. These data were additionally verified in the hospital's patient data system. The questionnaire was completed by one or both parents of the examined children.

### 2.3. Statistical Analysis

For statistical analysis, the following descriptive measures were calculated: mean, standard deviation (SD), median, and the first and third quartiles (Q1–Q3). Compliance with the normal distribution of quantitative variables was checked via the Shapiro–Wilk test. The Mann–Whitney *U*-test was conducted to examine differences in quantitative or ordinal variables between the T1D and control groups. Differences in qualitative variables between groups were checked using a chi-square test. Statistical analyses were performed using PS IMAGO PRO 7 (IBM SPSS Statistics 27, Armonk, NY, USA). The level of statistical significance was set at *p*  < 0.05.

## 3. Results

### 3.1. Characteristics of the Study Population

The characteristics of patients with T1D and the comparison group are presented in Table 1.

### 3.2. Overall Taste Test Results

The median total taste score (Me) for all subjects was 12.0 (10.0–13.0). The total scores were lower in the T1D group, Me = 11 (11.0–13.0), compared to the control group, Me = 12 (10.0–13.0). However, no statistically significant differences were found between the two groups (*p*=0.9470). There was, however, a significant difference between the groups in the sweet taste test. High values were shown in the T1D group. Median scores for sweet taste were 4.0 (3.0–4.0). In the control group, the median was lower and amounted to 3.0 (3.0–4.0), (*p*=0.0001). The mean score in the T1D group was (mean ± SD: 3.48 ± 0.60), and in the control group it was (mean ± SD: 3.15 ± 0.69). In the case of the other taste tests (bitter, salty, and sour), it was no differences between the groups. Median scores for the individual taste qualities were in T1D and control group as follows: sour taste (median ± Me = 2.0 [2.0–3.0] and median ± Me = 3.0 [2.0–3.0], *p*=0.4031); salty taste (median ± Me = 3.0 [2.0–4.0] and median ± Me = 3.0 [2.0–4.0], *p*=0.7831); and bitter taste (median ± Me = 3.0 [2.0–4.0] and Me = 3.0 [2.0–4.0], *p*=0.5191) (Table 2).

### 3.3. Effect of Diabetes Duration

The division of the research group into three groups according to the duration of diabetes (1–4, 5–9 years, and 10 and more) showed no significant differences in taste test results. We found more differences comparing the results of sensory tests of children with T1D for more than 10 years with the control group. In the T1D group for more than 10 years, median scores for bitter taste amounted to Me = 3.0 (2.0–4.0) and were lower than in the control group, Me = 3.0 (3.0–4.0), (*p*=0.0380). The mean score in the T1D group was (mean ± SD: 2.54 ± 1.02), and in the control group it was (mean ± SD: 2.94 ± 1.01). Statistically significant differences between the two groups were also found in the case of sweet taste. In the T1D group, median scores for sweet taste amounted to Me = 3.0 (2.0–4.0) and were higher than in the control group, Me = 3.0 (1.0–4.0), (*p*=0.0311). The mean score in the T1D group was (mean ± SD: 3.43 ± 0.60), and in the control group it was (mean ± SD: 3.15 ± 0.69) (Table 3).

### 3.4. Hypogeusia Result

In 42 (28.37%) adolescents with T1D, the results of taste tests signified a hypogeusia. Most of them, as many as 17 persons, had negative results of sour taste recognition (score lower than 2). Only 4% of the children were able to correctly identify all four sour taste strips. In the control group, hypogeusia was found in 24 (24.00%) adolescents. Non-normative results in the T1D group in boys were 23 (31.08%) and in girls were 19 (25.67%), and in the control group in boys were 15 (27.27%) and in girls were nine (20.00%). The results in both study groups were similar.

### 3.5. PROP Test Result

In total, 158 children (63.7%) were PROP tasters. 90 (36.3%) participants reported their feeling as having no sensation or reported that the strip “tastes like paper, eventually felt something, but could not qualify or define their sensations correctly. In the T1D group, there were 66.2% PROP tasters, while in the control group, there were 66% PROP tasters. There were no significant differences in the perception of the PROP factor between T1D and the control group (Table 2).

### 3.6. *U*-Sniff Test Result

In the control group most of the children, *n* = 87 (87.0%), showed normative results in the *U*-Sniff test. This means that their scores were equal to or greater than 10. Thirteen children had scores below 10, which means hyposmia, which is a partial loss of the sense of smell [20].

In the T1D group, a score lower than 10 was obtained by 32 teenagers (21.62%). This means that the normative result was achieved by almost 79% of the respondents. In both groups, there were no values below 7, which means that there were no people with a significant loss of olfactory sensitivity.

We found no significant differences between T1D and the control group of the *U*-Sniff test. Median for both groups was the same: Me = 11.0 (10.0–12.0), (*p*=0.6399). The mean score in the T1D group was (mean ± SD: 10.62 ± 1.38), and in the control group it was (mean ± SD: 10.79 ± 1.10).

However, a comparison of the results of teenagers with diabetes for over 10 years with the control group shows a significant difference in the *U*-Sniff test results. Lower results in the test were observed in the T1D group (median ± Me = 10 [9.0–12.0]) compared to the control group (median ± Me = 11 [10.0–12.0], *p*=0.0370) (Table 3).

**Figure 1:**
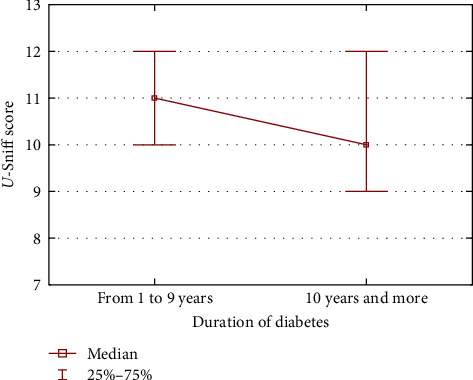
Olfactory function depending on the duration of diabetes.

The effect of disease duration is also significant when comparing test results for children with diabetes of 10 years of duration and children with diabetes of less than 10 years duration. Children with shorter-term diabetes obtained significantly higher results than children with longer-lasting diabetes, respectively (median ± Me = 11.0 [10.0–12.0] vs. median ± Me = 10 [9.0–12.0], *p*=0.0408) (Figure 1).

### 3.7. Association of Taste and Smell Recognition With Personal and Health Parameters in T1D Participants

Significant differences were found in taste test results in the T1D group with CD. As reported in Table 4, the results for recognizing sweet, sour, bitter tastes, and the overall taste score were significantly lower than in other teenagers with T1D. Overall test scores differed significantly, in the T1D group with CD median ± Me = 10.0 (9.0–11.0) vs. in the T1D group median ± Me = 12.0 (11.0–13.0), (*p* ≤ 0.0001). Significantly lower values were also found for a bitter taste test, respectively (median ± Me = 2.0 [1.0–3.0] vs. median ± Me = 3.0 [2.0–4.0], *p*=0.0004). Slightly lower but still significant differences were found in the case of sour and sweet taste results. For sour taste, respectively (median ± Me = 2.0 [2.0–2.0] vs. median ± Me = 2.0 [2.0–3.0], *p*=0.0437). In case sweet taste results were (median ± Me = 3.0 [3.0–4.0] vs. median ± Me = 4.0 [3.0–4.0], *p*=0.0488).

In the group of young patients with diabetes, girls achieved significantly higher results in the overall taste test (median ± Me: 12.0 [11.0–13.0]) compared to boys (median ± Me: 11.0 [10.0–13], *p*=0.0425). Girls' test results were also significantly higher in the case of bitter taste (median ± Me: 3.0 [2.0–4.0]) compared to boys (median ± Me: 3.0 [2.0–3.0], *p*=0.0065). In the *U*-Sniff test, the group of girls also showed significantly higher results (median ± Me: 11.0 [10.0–12.0]) compared to boys (median ± Me: 10.0 [9.0–12.0], *p*=0.0129). There were also no correlations between test and smell results and other personal variables (including BMI, breastfeeding, or presence of diabetes in the immediate family) (Table 5).

## 4. Discussion

A healthy lifestyle is important for children with T1D to maintain health, prevent cardiovascular disease, and control blood glucose levels. A healthy lifestyle primarily involves eating a balanced diet and getting regular exercise. Tracking carbohydrate intake, either through carb counting or estimation based on experience, is key to achieving optimal blood sugar levels [25]. Taste and smell sensitivity play a major role in food acceptance. However, how this relates to people's final food choices is still debatable [7, 26]. Moreover, there is growing interest in understanding taste perception's role in satiety, energy balance, and long-term health [27]. Taste dysfunction in diabetes can hinder dietary adherence, leading to negative effects on glycemic control [28]. The number of studies on taste sensitivity in individuals with T1D is limited. To date, the majority of research on this topic has focused on adult populations. For adults, especially after the age of 60, there is a decline in taste and smell function as part of the normal aging process [29]. In the case of diabetic patients, the taste may also change as a consequence of possible disease-related complications. However, it should be noted that some information regarding sensory sensitivity and T2D may not be fully applicable to T1D. Catamo et al. [30] reported that the incidence of taste and smell ID disorders was higher in T2D patients compared to healthy controls, and a possible relationship with glycemic levels emerged. The development of taste impairment in T2D has also been related to both microvascular and macrovascular issues caused by the disease [31, 32]. Yazla et al. [5] confirm that T2D is associated with olfactory and gustatory dysfunctions. The fact that there was no difference between diabetic patients with and without DPN suggests central neuropathy. Hyperglycemia and its association with microvascular complications such as polyneuropathy may be one of the factors that may affect taste disorders in diabetes. There are also reports in which people with diabetes without polyneuropathy, taste impairment was also revealed [33]. The taste dysfunction seen in T1D could also be caused by inflammation of the oral mucosa or decreased salivation [34, 35].

In this paper, we compared taste recognition between young people with T1D and healthy people. We did not find a reduced overall taste perception or bitterness in PROP participants with T1D compared to healthy participants. Available data on perceptions of basic tastes in T1D are limited and contradictory. Some older studies showed a difference in general taste perception between diabetics and healthy adults [6, 36], while others, slightly more recent, report no differences [37–39]. According to recent studies conducted on children and adolescents, young diabetics appear to have a comparatively lower ability to recognize taste characteristics than the control group. It was also noted that young diabetics exhibited lower sensitivity towards individual tastes of bitter and sour. In Mameli et al.'s study [40], the taste strips method was used, as in our case. However, the study raises doubts due to the small research group. The diabetic group exhibited lower overall taste perception compared to the control group, primarily due to their scores in the bitter and sour taste tests, which were significantly lower. In the Catamo et al. study [41], similar relationships were found, that is, significantly reduced overall taste perception, PROP bitterness, and sourness in citric acid participants with T1D. It is worth noting that the methodology of taste testing was different. In the comparative study, one concentration of each taste was used. In our study, the participants were asked to detect four different concentrations of each taste. Some previous studies have shown that women in the diabetic group, as well as in the general population, have a greater ability to correctly identify tastes [42, 43]. In our study, we confirmed a similar relationship among teenagers. Girls with T1D showed significantly higher overall taste test scores compared to boys (mean ± SD of 11.80 ± 2.10 vs. mean ± SD of 11.12 ± 2.23, *p*=0.0425). Significantly higher results in girls with T1D were also found for bitter taste (mean ± SD of 3.10 ± 1.00) compared to boys with T1D (mean ± SD of 2.59 ± 1.08, *p*=0.0065). It should be noted that girls from T1D had significantly higher results than boys also in the olfactory test (mean ± SD of 10.90 ± 1.12 vs. mean ± SD of 10.31 ± 1.48, *p*=0.0129).

Interestingly, our study revealed differences between diabetics with longer-term disease (≥10 years) and healthy controls. The differences concerned the sweet taste, the bitter taste, and the results of the *U*-Sniff test. The bitter taste test results obtained by diabetics were significantly lower, which confirms their lower sensitivity to bitter taste. Numerous studies indicate that greater sensitivity to the bitter taste in the form of propylthiouracil (PROP) is associated with generally increased taste sensitivity [44, 45]. In addition, the analysis of taste recognition by children using taste strips showed that children and adolescents with T1D more often correctly identified sweet tastes at lower sucrose concentrations compared to healthy adolescents. Similar conclusions regarding the sweet taste sensitivity of diabetics have appeared in several recent reports [5, 46]. Sińska et al. [47] observed that despite the higher hedonic score of solutions with a higher concentration of sweet taste in people with T1D than in healthy people, the result of the test assessing sweet taste perception in diabetics was higher than in healthy people. The greater sensitivity to sweet taste found in children and adolescents with T1D can be explained by adherence to dietary guidelines, which primarily involve restricting easily digestible carbohydrates, usually sweet-tasting products. People who regularly limit their sugar intake tend to have a heightened sensitivity to its taste. Healthy children tend to eat all types of foods and rarely monitor their consumption of sugary treats in their daily diet. Some studies clearly suggest that people who perceive products as sweeter have lower energy and carbohydrate intakes (starches, total sugars, fructose, and glucose), and less frequent intake of sugary foods and sugary drinks compared to those who perceive products as less sweet [48, 49]. According to Tan and Tucker [50], taste sensitivity is not seen as a reliable indicator of food intake. Taste exposures in tests may be completely different from those experienced during normal eating. However, positive associations have been shown between hedonic responses and food rich in sugar consumption.

In our study, we also assessed the olfactory function of diabetic and healthy adolescents. We noted that generally, the olfactory function of patients with T1D did not significantly differ from that of the control group, which corresponds to the results of numerous prior studies, most of which focused on adults [19, 37, 38, 51–53]. Within this study, healthy children scored a mean ± SD of 10.79 ± 1.10 points out of 12. Children with T1D group obtained a similar value (mean ± SD of 10.62 ± 1.38). However, the duration of diabetes appears to be important. Adolescents diagnosed with diabetes for at least 10 years had significantly lower *U*-Sniff test scores (mean ± SD of 10.19 ± 1.49, *p*=0.0370). Most of the existing reports confirm the impaired olfactory function in both T2D [2, 3, 38, 54] and T1D [11, 37, 38, 54, 55], often linking them to diabetic complications, nephropathy [6, 56], or retinopathy and neuropathy [2, 3, 6, 11]. The main risk factor for the development of late complications of T1D, including neuropathy, is long-term glycemic disorders [57]. The probability of its occurrence increases with the duration of diabetes and, sometimes, the severity of hyperglycemia. Neuropathy can also occur suddenly as a result of hypoglycemia [58]. This complication should be considered in all patients with T2D and patients with T1D for at least 5 years [59]. In a study conducted in the UK, the average incidence of neuropathy among diabetic patients increased with the duration of diabetes. In patients treated for more than 10 years, the complication rate was 36.8%. Some reports suggest a higher incidence of diabetic neuropathy, reaching 54%–90% [60]. Therefore, it seems that decreased olfactory function may be a common symptom of emerging diabetic complications. The probability of complications increases with the duration of diabetes.

During the analyses, it turned out that the olfactory and taste test results in a small group of diabetic adolescents diagnosed with CD were interesting. CD occurs in patients with T1D, ranging a prevalence of 4.4%–11.1% [61]. The results of taste tests in this group of adolescents differ significantly from those of other diabetics. The taste test total scores were higher for the T1D group, Me = 12 (11.0–13.0), compared to the group of diabetics with CD, Me = 10 (9.0–11.0). The mean scores were (mean ± SD of 11.77 ± 1.77 vs. mean ± SD of 9.67 ± 2.12, *p* ≤ 0.0001), respectively. Lower overall test scores indicate lower taste sensitivity in children with CD. Significantly lower results in children with CD were found also for sweet taste (mean ± SD of 3.19 ± 0.68 vs mean ± SD of 3.53 ± 0.57, *p*=0.0488); sour taste (mean ± SD of 2.00 ± 0.71 vs. mean ± SD of 2.38 ± 0.87, *p*=0.0437); and bitter taste (mean ± SD of 2.00 ± 1.14 vs. mean ± SD of 2.98 ± 0.99, *p*=0.0004). It is known that CD is a specific disease that is associated with significant changes in diet. A significant part of gluten-free products has a changed composition, so their sensory properties are also changed. The ingredients of gluten-free food products are usually not fully known. The sparse data generally describe the composition of gluten-free foods as lower in protein, higher in fat, and lower in fiber than foods containing gluten. Moreover, it is indicated that gluten-free food may cause deficiencies of many nutrients, especially vitamin B, vitamin D, calcium, magnesium, iron, or zinc [62]. It is worth mentioning that an adequate supply of certain vitamins and minerals, especially zinc, is extremely important for the proper functioning of the sense of taste [63, 64]. Of course, zinc deficiency in children with CD is one of many factors that may affect taste perception. More sensory tests are also needed to confirm and explain differences in taste perception in children with CD.

## 5. Conclusion

Our results did not show statistical significant differences in the olfactory functions and taste perception of diabetics and healthy adolescents. However, the lowest test scores among adolescents with the longest duration of diabetes (>10 years) suggest that changes in sensory perception are related to the duration of diabetes. Unfavorable changes may concern both the perception of individual tastes, for example, sweet or bitter, and a decrease in overall taste perception, as well as a decrease in olfactory functions. Sensory assessment in clinical practice in children and adolescents with diabetes is currently important in monitoring dietary habits. In the future, it may also be useful in diagnosing diabetes complications.

## 6. Strengths and Limitations of the Study

The study presents certain inherent risks of bias, primarily due to the stronger interest in obtaining accurate responses from participants as opposed to the general population. It is noteworthy that children diagnosed with diabetes exhibited favorable results, likely because the assessments were conducted during their visits to the diabetes specialist in a hospital setting. Conversely, the control group participated in nonbinding evaluations aimed at assessing their senses of taste and smell.

Furthermore, the established cut-off criterion for hyposmia appears to be somewhat arbitrary, as the prevalence of reduced olfactory function among children has not been thoroughly documented. To enhance the precision of olfactory function evaluations, it is advisable to consider implementing more comprehensive testing methods or assessments. Lastly, the study is limited by its reliance on a single center, the application of single blinding, and the absence of complete randomization.

This study presents several notable advantages, primarily its substantial sample size. In contrast to prior research on sensory tests among children with T1D, which has frequently involved smaller participant groups, this investigation offers a more robust analysis. Furthermore, the study includes the evaluation of both taste and smell, as well as the incorporation of a control group. It is also worth mentioning that very little research has been done on this topic.

## Figures and Tables

**Table 1:** Characteristics of the study sample.

Features	T1D group	Control group	*p*-Value
*N*	148	100	—
Age (years)^a^	13 (11, 15)	13 (12, 14)	0.6100^b^
Gender (male/female)	74/74	55/45	0.4394^c^
Height^a^	158 (149, 166)	158 (149, 165)	0.8546^b^
Weight^a^	50.3 (40.4, 57.4)	47.6 (37.7, 58.9)	0.5691^b^
BMI (percentile grids)			
<5%tile (underweight; %)	1 (0.7)	0 (0.0)	—
5–85%tile (normal weight; %)	120 (80.1)	77 (77.0)	0.6199^c^
>85%tile (overweight or obesity; %)	27 (18.2)	23 (23.0)	—
Total fat content (%)^a^	15.4 (12.9, 23.9)	20.4 (14.8, 30.3)	<0.0001^b^
T-Chol (mg/dL)^a^	172 (151, 193)	—	—
LDL-Chol (mg/dL)^a^	98 (82, 116)	—	—
HDL-Chol (mg/dL)^a^	59 (52, 69)	—	—
TG (mg/dL)^a^	71.5 (55, 103)	—	—
HBAIc (%)^a^	7.02 (6.46, 7.71)	—	—

^a^Median with interquartile range.

^b^ *U* Mann–Whitney test.

^c^χ^2^ test.

**Table 2:** Taste sensitivity and *U*-Sniff score in T1D group and control group.

Features	T1D group	Control group	*p*-Value
PROP tasters (%)	92 (62.2)	66 (66.0)	0.5375^c^
PROP nontasters (%)	56 (37.8)	34 (34.0)	0.5375^c^
Sensitivity to sweet taste^a^	4.0 (3.0, 4.0)	3.0 (3.0, 4.0)	0.0001^b^
Sensitivity to salty taste^a^	3.0 (2.0, 4.0)	3.0 (2.0, 4.0)	0.7831^b^
Sensitivity to sour taste^a^	2.0 (2.0, 3.0)	3.0 (2.0, 3.0)	0.4031^b^
Sensitivity to bitter taste^a^	3.0 (2.0, 4.0)	3.0 (2.0, 4.0)	0.5191^b^
Sensitivity to all taste^a^	11 (11, 13)	12 (10, 13)	0.9470^b^
*U*-Sniff test (odor test)^a^	11 (10, 12)	11 (10, 12)	0.6399^b^

^a^Median with interquartile range.

^b^ *U* Mann–Whitney test.

^c^χ^2^ test.

**Table 3:** Taste sensitivity and *U*-Sniff score in adolescents with diabetes for at least 10 years and control group.

Features	T1D (10 years) group	Control group	*p*-Value
*N*	37	100	—
Sensitivity to sweet taste^a^	3.0 (2.0, 4.0)	3.0 (1.0, 4.0)	0.0311^b^
Sensitivity to salty taste^a^	3.0 (3.0, 4.0)	3.0 (3.0, 4.0)	0.6561^b^
Sensitivity to sour taste^a^	2.0 (2.0, 4.0)	2.0 (2.0, 4.0)	0.2988^b^
Sensitivity to bitter taste^a^	3.0 (2.0, 4.0)	3.0 (3.0, 4.0)	0.0380^b^
Sensitivity to all taste^a^	11.0 (9.0, 13.0)	12.0 (10.0, 13.0)	0.3126^b^
*U*-Sniff test (smell test)^a^	10.0 (9.0, 12.0)	11.0 (10.0, 12.0)	0.0370^b^

^a^Median with interquartile range.

^b^ *U* Mann–Whitney test.

**Table 4:** Taste sensitivity and *U*-Sniff score in T1D group with celiac disease.

Features	Celiac group	T1D group	*p*-Value
*N*	21	127	—
Sensitivity to sweet taste^a^	3.0 (3.0, 4.0)	4.0 (3.0, 4.0)	0.0488^b^
Sensitivity to salty taste^a^	2.0 (2.0, 4.0)	3.0 (2.0, 4.0)	0.1661^b^
Sensitivity to sour taste^a^	2.0 (2.0, 2.0)	3.0 (2.0, 3.0)	0.0437^b^
Sensitivity to bitter taste^a^	2.0 (1.0, 3.0)	3.0 (2.0, 4.0)	0.0004^b^
Sensitivity to all taste^a^	10.0 (9.0, 11.0)	12.0 (11.0, 13.0)	~0.001^b^
*U*-Sniff test (smell test)^a^	10.0 (9.0, 11.0)	11.0 (10.0, 12.0)	0.1246^b^

^a^Median with interquartile range.

^b^ *U* Mann–Whitney test.

**Table 5:** Taste sensitivity and *U*-Sniff score results by gender, BMI, breastfeeding, and the presence of diabetes (TD) in immediate family.

Features	Girls with T1D	Boys with T1D	*p*-Value
*N*	74	74	—
Sensitivity to sweet taste^a^	4.0 (3.0, 4.0)	3.0 (3.0, 4.0)	0.2293^b^
Sensitivity to salty taste^a^	3.0 (2.0, 4.0)	3.0 (2.0, 4.0)	0.7128^b^
Sensitivity to sour taste^a^	3.0 (2.0, 3.0)	2.0 (2.0, 3.0)	0.3122^b^
Sensitivity to bitter taste^a^	3.0 (2.0, 4.0)	3.0 (2.0, 3.0)	0.0065^b^
Sensitivity to all taste^a^	12.0 (11.0, 13.0)	11.0 (10.0, 13.0)	0.0425^b^
*U*-Sniff test (odor test)^a^	11.0 (10.0, 12.0)	10.0 (9.0, 12.0)	0.0129^b^

	**BMI <85th (%tile)**	**BMI ≥85th (%tile)**	***p*-Value**

*N*	121	27	—
Sensitivity to sweet taste^a^	3.0 (3.0, 4.0)	4.0 (3.0, 4.0)	0.0948^b^
Sensitivity to salty taste^a^	3.0 (2.0, 4.0)	3.0 (2.0, 3.0)	0.5267^b^
Sensitivity to sour taste^a^	2.0 (2.0, 3.0)	2.0 (2.0, 3.0)	0.7507^b^
Sensitivity to bitter taste^a^	3.0 (2.0, 4.0)	3.0 (2.0, 4.0)	0.8659^b^
Sensitivity to all taste^a^	11.0 (10.0, 13.0)	12.0 (10.0, 13.0)	0.9466^b^
*U*-Sniff test (odor test)^a^	11.0 (10.0, 12.0)	11.0 (10.0, 12.0)	0.4028^b^

	**Breastfeeding**	**Not breastfeeding**	***p*-Value**

*N*	32	116	—
Sensitivity to sweet taste^a^	3.0 (3.0, 4.0)	4.0 (3.0, 4.0)	0.7714^b^
Sensitivity to salty taste^a^	3.0 (2.5, 4.0)	3.0 (2.0, 4.0)	0.1320^b^
Sensitivity to sour taste^a^	2.5 (2.0, 3.0)	2.0 (2.0, 3.0)	0.5558^b^
Sensitivity to bitter taste^a^	3.0 (2.0, 3.5)	3.0 (2.0, 4.0)	0.8430^b^
Sensitivity to all taste^a^	11.5 (11.0, 13.5)	11.0 (10.0, 13.0)	0.2443^b^
*U*-Sniff test (odor test)^a^	11.0 (9.5, 12.0)	11.0 (10.0, 12.0)	0.8074^b^

	**TD in the family**	**No TD (family)**	***p*-Value**

*N*	53	95	—
Sensitivity to sweet taste^a^	3.0 (3.0, 4.0)	4.0 (3.0, 4.0)	0.1237^b^
Sensitivity to salty taste^a^	3.0 (2.0, 3.0)	3.0 (2.0, 4.0)	0.2201^b^
Sensitivity to sour taste^a^	2.0 (2.0, 3.0)	2.0 (2.0, 3.0)	0.6949^b^
Sensitivity to bitter taste^a^	3.0 (2.0, 4.0)	3.0 (2.0, 4.0)	0.9565^b^
Sensitivity to all taste^a^	11.0 (10.0, 13.0)	12.0 (10.0, 13.0)	0.1824^b^
*U*-Sniff test (odor test)^a^	11.0 (9.0, 12.0)	12.0 (10.0, 12.0)	0.9027^b^

^a^Median with interquartile range.

^b^ *U* Mann–Whitney test.

## Data Availability

The data that support the findings of this study are available from the corresponding author upon reasonable request.
